# COSMIC: the catalogue of somatic mutations in cancer

**DOI:** 10.1186/gb-2011-12-s1-p3

**Published:** 2011-09-19

**Authors:** Nidhi Bindal, Simon A Forbes, David Beare, Prasad Gunasekaran, Kenric Leung, Chai Yin Kok, Mingming Jia, Sally Bamford, Charlotte Cole, Sari Ward, Jon Teague, Michael R Stratton, Peter Campbell, Andrew P Futreal

**Affiliations:** 1 Wellcome Trust Sanger Institute, Cambridge, UK

## 

The Catalogue Of Somatic Mutations In Cancer (COSMIC) [1] is one of the largest repositories of information on somatic mutations in human cancer. The project has been running for more than ten years as part of the Cancer Genome Project (CGP) at the Wellcome Trust Sanger Institute in the UK.

The data in COSMIC are curated from a variety of sources, primarily the scientific literature and large international consortia. The project includes information from the CGP, along with data from other consortia such as the International Cancer Genome Consortium and The Cancer Genome Atlas. In addition, COSMIC is regularly updated with the genes highlighted in the Cancer Gene Census, which curates the scientific literature for known cancer genes [2].

With the advent of whole exome and genome sequencing technology, the amount of data in COSMIC is increasing rapidly. The recent COSMIC release (version 53; 18 May 2011) contains 608,042 tumor and cell line samples, annotating 176,856 mutations across 19,439 genes, with 352 full exomes, 43 whole genome rearrangement screens and 4 full genomes now available. The data are updated regularly, with new releases scheduled every two months.

COSMIC provides a large number of graphical and tabular views for interpreting and mining the large quantity of information, as well as the facility to export the relevant data in various formats. The website can be navigated in many ways to examine mutation patterns on the basis of genes, samples and phenotypes, which are the main entry points to COSMIC.

COSMIC also provides various options to browse the data in a genomic context. Integration with the Ensembl genome browser allows the visualization of full genome annotations, together with COSMIC data, on the GRCh37 genome coordinates. COSMIC also contains its own genome browser, which facilitates data analysis by combining genome-wide gene structures and sequences with rearrangement breakpoints, copy number variations and all somatic substitutions, deletions, insertions and complex gene mutations.

The main COSMIC website [1] encompasses all of the available data. However, within COSMIC, the Cancer Cell Line Project [3] is a specialized component, which provides details of the genotyping of almost 800 commonly used cancer cell lines, through the set of known cancer genes. Its focus is to identify driver mutations, or those likely to be implicated in the oncogenesis of each tumor.

This information forms the basis for integrating COSMIC with the Genomics of Drug Sensitivity in Cancer project [4], which is a joint effort with the Massachusetts General Hospital [5] to screen this panel of cancer cell lines against potential anticancer therapeutic compounds to investigate correlations between somatic mutations and drug sensitivity.

Data on somatic mutations in cancer are being produced at a rapidly increasing rate, and the combined analysis of large distributed datasets is becoming ever more difficult. However, COSMIC curates and standardizes this information in a single database, providing user-friendly browsing tools and analytical functions, thus ensuring its role as a key resource in human cancer genetics.
